# Clinical and radiological results of the vascularized medial femoral condyle graft for scaphoid non-union

**DOI:** 10.1007/s00402-020-03386-7

**Published:** 2020-03-02

**Authors:** Marco Keller, Tobias Kastenberger, Anizar Faizi Anoar, Peter Kaiser, Gernot Schmidle, Markus Gabl, Rohit Arora

**Affiliations:** 1 Department of Trauma Surgery, Medical University of Innsbruck, Anichstrasse 35, 6020 Innsbruck, Austria; 2 Department of Orthopaedics and Traumatology, Kantonsspital Baselland, Rheinstrasse 26, 4410 Liestal, Switzerland; 3 Department of Orthopaedics and Traumatology, Kuala Lumpur Hospital, Jalan Pahang, 50586 Kuala Lumpur, Malaysia

**Keywords:** Vascularized, Femoral condyle, Graft, Scaphoid, Non-union, Scaphoid fracture

## Abstract

**Introduction:**

This study evaluated the use of a free vascularized bone graft with and without cartilage from the medial femoral condyle (MFC) in patients with recalcitrant scaphoid non-union, with a special focus on union rates and the osteochondral graft for proximal pole destruction.

**Materials and methods:**

Thirty-eight avascular scaphoid non-unions in 37 patients who were treated with a free osteoperiosteal or osteochondral MFC graft were retrospectively evaluated (mean follow-up 16 months). Bone union, the scapholunate and the radiolunate angles were evaluated on X-ray images. The range of motion, grip strength, VAS, DASH and PRWE scores were evaluated clinically.

**Results:**

The overall union rate was 95%. Bone union was achieved in 27 out of 29 (93%) scaphoids treated with a free osteoperiosteal MFC grafts and in 9 out of 9 (100%) scaphoids treated with a free osteochondral MFC graft. The range of motion remained almost unchanged, while grip strength increased significantly (34 kg vs. 44 kg) and the VAS (22–5), DASH (59–19) and PRWE (62–30) score decreased significantly. The scapholunate (71°–65°) and radiolunate (28°–18°) angle decreased. No major donor site morbidity was observed. Postoperative complications were observed in eight cases (21%).

**Conclusions:**

The vascularized medial femoral bone graft leads to a good functional outcome in the treatment of scaphoid non-unions. The graft provides adequate blood supply and structural stability to the scaphoid. A proximal pole destruction can be replaced using an osteochondral graft with promising short-term results preventing carpal osteoarthritis and collapse.

## Background

Owing to its frail blood supply, the scaphoid shows an increased susceptibility to the development of a posttraumatic non-union with consecutive progressive carpal instability after fracture. Healing of a scaphoid fracture is especially critical and prolonged in proximal pole fractures (average healing time for fractures of the proximal third of the scaphoid 113 days, for waist fractures 65 days and for distal pole fractures 53 days) [15]. Langer et al. recently postulated that in a posttraumatic humpback deformity, only the palmar edges of the proximal and distal fragment touch each other. This insufficient contact of the fragments might be another cause of the increased rate of posttraumatic non-union in the scaphoid [26].

Treatment of scaphoid non-union typically consists of surgical treatment using a bone graft for better healing potential. Despite increasing controversy about whether to use a vascularized or avascularized bone graft, some studies in the past suggested that to improve bone healing, for some indications, the use of a vascularized bone graft can lead to a more favorable outcome than the use of an avascularized bone graft [29]. Traditionally accepted indications for a vascularized bone graft at the scaphoid are initially failed attempts of fracture fixation with established scaphoid non-union, non-unions that have not united after a non-vascularized bone grafting, not treated non-unions following overseen fractures and avascular bone necrosis (Morbus Preiser) [12, 39, 45, 46]. Schmidle et al. recently proposed a more elaborated treatment algorithm with the addition of trabecular structure, sclerosis and proximal fragment fragmentation in 2D-CT data and potential blood supply based on the location of the non-union in 3D-CT reconstructions. They found out that proximal fragments with preserved trabecular structure without fragmentation show a statistically significant higher bone healing capacity. If, in addition, patients showed no sclerosis, the time to union was also significantly shorter. Thus, the derived algorithm could facilitate decision-making, but has still to be proven viable in routine use [42].

Munk et al. performed a systematic review of the literature regarding scaphoid non-union and reported an average union rate of 78% for proximal pole scaphoid non-union, including those with avascular necrosis (AVN) that were treated with conventional non-vascularized bone grafts [30]. With the improvement of microsurgical techniques in hand surgery, the use of free vascularized bone grafts increased reported healing rates up to 100% [4, 8, 9, 21, 22].

Several different vascularized bone grafts exist which can be used [1, 13, 23, 25, 27, 38, 41, 44]. One of them is a free vascularized graft from the medial femoral condyle (MFC), which can be harvested as an osteoperiosteal or osteochondral graft.

Data on the outcome of this graft, especially on the osteochondral graft for proximal pole destruction, is rare.

Therefore, this study evaluated the use of a free vascularized bone graft with and without cartilage from the MFC in patients with recalcitrant scaphoid non-union. A special focus was set on the osteochondral graft for proximal pole destruction and union rates.

## Patients and methods

All patients who underwent surgical treatment of scaphoid non-unions using a free vascularized osteoperiosteal or osteochondral MFC graft between April 2008 and March 2016 at our institution were identified and included. A total of 38 wrists in 37 patients (36 male, 1 female) were reviewed. The mean age at the time of surgery was 26.2 years (range 17–42 years). The right wrist was affected in 19 (51%) patients and the left one in 17 (46%) patients. One patient had surgical treatment on both wrists (3%). Mean time of persisting non-union until the surgery was 51 months (range 2–184 months) and mean follow-up time was 16 months (range 6–53 months).

The indication for a vascularized MFC bone graft was based on patient’s symptoms and the correlation with preoperative radiological findings. All patients complained of wrist pain, reduced grip strength and/or reduced range of wrist motion. In all cases, preoperative CT scans showed an established non-union with a proximal pole fragment associated with bone resorption and cystic changes at the fracture site. A preoperative gadolinium-enhanced MRI examination was available for 31 (84%) patients. In all of those, either a compromised or completely missing perfusion of the proximal fragment was seen.

**Fig. 1:**
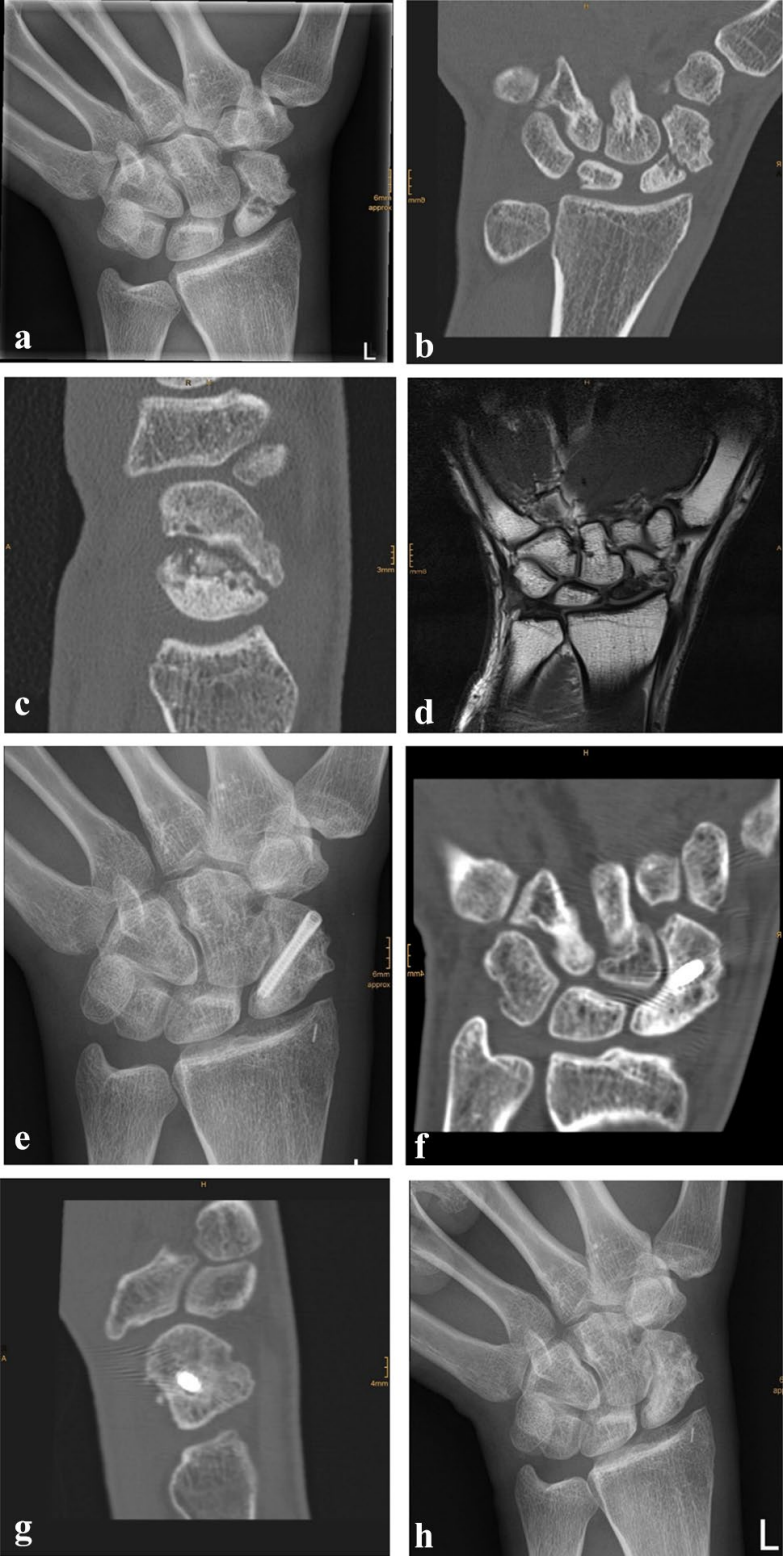
**a** Dorsopalmar radiograph of a 31-year-old man showing a non-union 3 years after sustaining a scaphoid fracture which initially remained undetected. **b** Coronal CT scan showing the scaphoid non-union in the mid-waist. **c** Sagittal CT scan. **d** Coronal gadolinium-enhanced MRI scan showing an avascular necrosis of the proximal pole. **e** Dorsopalmar radiograph 2 years after implantation and screw fixation of a vascularized bone graft from the medial femoral condyle showing complete healing with no signs of carpal malalignment. **f** Coronal CT scan showing complete healing. **g** Sagittal CT scan. **h **Dorsopalmar radiograph after removal of the screw (2.5 years after surgery) showing no signs of osteoarthritis or carpal collapse

**Fig. 2:**
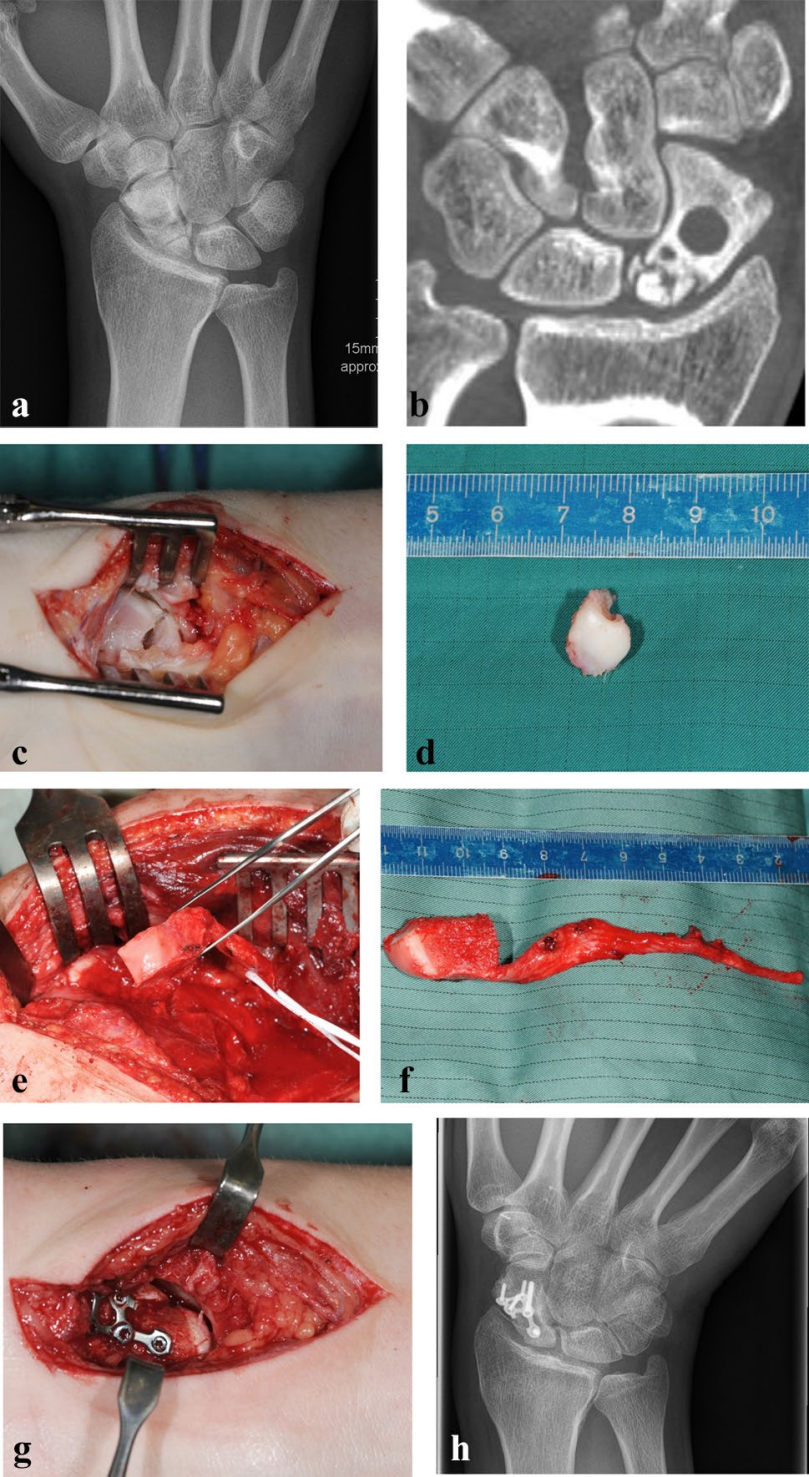
**a** Dorsopalmar radiograph of a 21-year-old man showing an established scaphoid non-union of the proximal pole. **b** Coronal CT scan showing the fragmented, necrotic proximal pole. **c** Intraoperative view showing the fragmented, necrotic proximal pole. **d** Intraoperative view showing the excised main proximal pole fragment. **e** Intraoperative view showing the harvesting of a osteochondral medial femoral condyle graft. **f** Intraoperative view showing the harvested osteochondral graft with a long pedicle. **g** Intraoperative view showing the replacement of the proximal pole with a osteochondral medial femoral condyle graft and plate fixation. **h** Dorsopalmar radiograph (3 years after surgery) showing the healed proximal pole without signs of carpal collapse

Due to a fragmented, unstable and avascular proximal pole or previously antegrade screw fixation with destruction of the proximal pole, 9 (24%) out of the 38 scaphoids were treated with a free vascularized osteochondral MFC graft and the remaining 29 (76%) scaphoids were treated using a free vascularized osteoperiosteal MFC graft. Two exemplary patients (1 × osteoperiosteal and 1 × osteochondral case) are shown in Figs. 1 and 2.

### Radiological assessment

Standard dorsopalmar and lateral radiographs of the wrist were taken.

Assessment was performed by a reviewer who was not involved in the treatment and was blinded to the functional outcome but not to the method of treatment. The pre- and postoperative radiographs were assessed for bone healing, the scapholunate and the radiolunate angle. Changes in the scapholunate angle were evaluated to assess palmar rotation of the scaphoid. The presence of a dorsal intercalated segment instability (DISI) was assessed by changes in the radiolunate angle on lateral radiographs. All radiological parameters were compared to the uninjured contralateral side. In cases with any doubt about the signs of bone healing, CT scans were performed.

### Functional assessment

Functional examination of all patients was performed by one single examiner who was not involved in the surgical treatment. Active range of motion (ROM) and grip strength were measured in a standardized manner for both wrist joints with a digital analysis system (Biometrics Ltd. E-LINK, Newport, UK). Final functional results were compared to the preoperative measurements and to the uninjured, contralateral side. Wrist pain was evaluated using a visual analog scale (VAS 0 = no pain, 100 = severe pain). Functional subjective outcome was assessed using the Disability of the Arm, Shoulder, and Hand (DASH) Questionnaire. It has a 0–100 point scale with 0 representing the best result. Additionally, at final follow-up the Patient-Rated Wrist Evaluation Score was recorded. This scale ranges between 0 and 150 points, with no points indicating an asymptomatic wrist.

### Surgical technique and postoperative treatment

The procedure was performed in the same manner as elaborately described earlier by Iorio, Bürger and Higgins and was usually conducted with a team of two to four surgeons [3, 17, 20]. A single palmar approach was performed in 27 patients and an additional dorsal approach was necessary in 11 patients. A dorsal approach was needed either to remove the previously inserted antegrade screw or to perform an end to side anastomosis of the bone graft artery to the second metacarpal artery, as the pedicle of the MFC was located on the dorsal side. The graft was fixed with a 1 mm K-wire in 22 cases, with a headless compression screw in seven cases and with a palmar locking scaphoid plate in nine patients.

A standardized aftercare protocol was followed with an above elbow cast fixation for 4 weeks (to minimize rotation forces on the wrist and the pedicle in the early postoperative phase during forearm rotation), followed by a below elbow immobilization for another 6 weeks with K-wire removal thereafter. The palmar plate was removed in two cases after a mean of 8.5 months because of disturbing impingement. The other patients with palmar locking plates (which was intraoperatively sometimes shortened or adapted) showed no complaints which would imply implant removal. The immobilization period was followed by passive and assisted active wrist exercises.

### Statistical analysis

Descriptive data are reported using means, standard deviations and the range. A paired Student’s *t* test was used for statistical analysis. Statistical significance was set at *p* < 0.05.

## Results

### Radiological results

Differentiated union rates with and without previous surgery are presented in Table 1.

**Table 1:** Differentiated union rates achieved in patients with and without previous surgery and with different surgical approaches

	Number of treated scaphoid non-unions	Union rate
All MFC grafts	38	36 (95%)
Osteochondral MFC grafts	9	9 (100%)
No previous surgery	23	23 (100%)
Previous screw fixation	6	4 (67%)
Previous bone grafting	9	9 (100%)
Volar approach	27	27 (100%)
Combined approach	11	9 (82%)

All patients completed the follow-up protocol. Bone union was achieved in 36 treated scaphoids (95%; 27 osteoperiosteal and nine osteochondral MFC grafts). The mean time to union was 16 weeks (range 12–22 weeks) for the patients treated with an osteoperiosteal MFC graft and 18 weeks (range 14–26 weeks) for the patients treated with an osteochondral graft. One patient (3%) achieved partial union (a residual gap of 1 mm was visible palmarly after 1 year in the CT scan). This patient was asymptomatic; therefore, no further treatment was performed. Two patients (5%) had persisting non-unions with the bone graft being continuously resorbed throughout the first postoperative year. One patient remained asymptomatic and denied further treatment. The other patient suffered from ongoing pain and restriction in the range of motion. This patient was treated 7 months after the free vascularized MFC graft with a free vascularized bone graft from the iliac crest that led to bone union after 4 months.

The humpback deformity and an associated DISI deformity were improved postoperatively and did not deteriorate during the follow-up. The scapholunate angle decreased by a mean of 6° and the radiolunate angle was partly restored with a mean of 10° improvement. Correction of the radiolunate angle was statistically significant (Table 2).

**Table 2:** Results of the scapholunate and radiolunate angle before surgery and at final follow-up of the injured and uninjured contralateral wrist (mean and standard deviation)

	Before surgery (SD)	Final follow-up (SD)	Uninjured contralateral side (SD)	*p* value (pre- vs. postoperatively)
All scaphoids (*n* = 38)				
Scapholunate angle (°)	71 (13.7)	65 (12.5)	57 (12)	0.29
Radiolunate angle (°)	28 (11)	18 (9)	9 (6)	0.01

The values of the final follow-up were compared to the preoperative values and contralateral side

### Functional results

The results for the range of motion and grip strength are shown in Table 3.

**Table 3:** Results of the range of motion and grip strength before surgery and at final follow-up of the injured and uninjured contralateral wrist (mean and standard deviation)

	Before surgery (SD)	Final follow-up (SD)	Uninjured contralateral side (SD)	*p* value (pre- vs. postoperatively)
All scaphoids (*n* = 38)				
Extension (°)	50 (12)	50 (15)	68 (12)	0.1
Flexion (°)	49 (11)	44 (16)	64 (11)	0.1
Grip strength (kg)	34 (8)	44 (13)	49 (10)	0.05

The values of the final follow-up were compared to the preoperative values and contralateral side

The mean pain level decreased significantly from 22 points (range 8–64) before surgery to five points (range 0–16) at final follow-up (*p* = 0.03). The mean preoperative DASH score was 59 points (SD 12) and decreased significantly to 19 points (SD 11) at final follow-up (*p* = 0.03). The mean PRWE score decreased significantly from 62 points (SD 10) before surgery to 30 points (SD 9) at final follow-up (*p* = 0.04). No patient had to change his/her occupation.

### Complications

Postoperative complications were observed in eight cases (21%) with seven (18%) being treated with subsequent surgical interventions. Loosened or dislocated implants which led to local irritation represented four out of those complications (one associated with hyperossification). All four implants were removed after an average of 14.5 months. One late surgical site infection was seen 3 months after surgery which was treated with removal of the metal implants (one K-wire), antibiotic therapy and a V.A.C. therapy (vacuum assisted closure) for 3 days. The patient then went to unproblematic bone union. One patient suffered from a venous bleeding that had to be surgically stopped on the second postoperative day. Another patient suffered from a postoperative functional Boutonniere-like malposition in the metacarpophalangeal joint of the thumb and was treated with surgical tenolysis and tendon transfer 7 months postoperatively. Finally, one patient suffered from delayed wound healing of the palmar approach to the scaphoid that eventually healed without surgical treatment.

No major donor site morbidity was observed. The only discomforts that patients noticed in the area of the medial femoral condyle were postoperative swelling, pain and hypesthesia in the area around the scar. All of these symptoms disappeared after 1 year or less.

## Discussion

This retrospective evaluation showed that the vascularized osteoperiosteal and osteochondral medial femoral condyle graft is a good option for the treatment of scaphoid non-unions showing a high union rate, reducing pain, preventing carpal collapse and improving carpal alignment and wrist function.

A special focus on the osteochondral graft even showed a 100% healing rate. Patients whose proximal pole is destroyed have few treatment options. If untreated, this would probably always lead to wrist destruction with consecutive osteoarthritis and collapse with increased pain and limited wrist function. In such cases, only salvage procedures like a radioscapholunate fusion, four-corner fusion or proximal row carpectomy seem imaginable. The findings of the present study showed that this subgroup of patients can have a good outcome if the proximal destroyed scaphoid pole is replaced with an osteochondral vascularized MFC graft. At our institution, the authors are confronted with a rather young and physically active population with a high functional demand. So when in doubt, the decision whether to try a reconstruction or conduct a salvage procedure would tend toward the attempt of reconstruction in most cases.

In the previous unsuccessful bone grafting (non-vascularized graft from the iliac crest), the authors also observed a union rate of 100% by using a vascularized MFC graft. The restoration of vascularity at the fracture site seems to have improved the healing potential in those cases.

Since pain levels decreased and functional scores increased significantly postoperatively, scaphoid non-unions should not be left untreated. Even the range of motion remained more or less the same despite a rather long postoperative cast fixation (4–6 weeks upper arm, then 4–6 weeks forearm) similar to Higgins et Buerger [17].

Previously failed screw fixation with or without bone grafting can be encouragingly treated with a vascularized MFC graft, leading to satisfied patients thereafter.

Earlier on, iliac crest bone grafts were used to treat scaphoid non-unions. Bone grafting in the classic Russe technique showed union rates of up to 92% [7, 14].

Other investigators found healing rates of 94% using avascular and 85–97% using vascularized iliac bone grafts [10, 36, 43]. Thus, the healing rate for the vascularized MFC graft in this study seems comparable to the iliac crest bone graft.

A long-term follow-up study also showed that 81% of the patients were pain free and the progression of osteoarthritis could be stopped in those patients who achieved bone healing [11]. The iliac bone crest therefore seemed to offer a reliable source for vascularized bone grafting to address scaphoid non-unions [2].

However, later surveys showed that despite the appealing union rate, the patients suffered from significant donor site morbidity. Harpf et al. followed a collective of 60 patients after scaphoid reconstruction with a vascularized iliac crest graft and found that 32% of the patients suffered from an impairment of the lateral femoral cutaneous nerve and 63% showed bone deformations at the donor site radiologically [16]. A systematic meta-analysis of 48 publications comparing (vascularized and non-vascularized) grafts from the distal radius and iliac crest for scaphoid non-union showed that both had similar union rates (89% vs. 87%), but that the iliac bone graft showed a significantly higher donor site morbidity [31].

In 1991, Sakai et al. first described the use of a new osteoperiosteal graft harvested from the medial condylar and supracondylar area of the femur, which was pedicled from the articular branch of the descending genicular artery. It was used on six patients with a fracture non-union of the upper extremity and all of them healed uneventfully after graft implantation [40]. After that, the graft was successfully used in non-unions of the upper limb [8] and the tibia [6]. The group of Kazuteru Doi were the first to describe the use of the vascularized femoral condyle graft in the scaphoid. The authors harvested the initially thin-layered, strictly osteoperiosteal graft with parts of cancellous bone. Ten patients with a scaphoid non-union were treated with this technique and all achieved union after an average of 12 weeks [9].

The following studies showed very promising results [4, 8, 22]. Jones et al. compared the distal radial pedicled vascular graft (1, 2-ICSRA-graft) to the free vascularized MFC graft in the treatment of patients with scaphoid waist non-unions with an avascular proximal pole and carpal collapse. In the present trial, the MFC graft (12 patients, 100% union rate) showed a considerably better outcome than the 1, 2-ICSRA-graft (ten patients, 40% union rate) [21]. However, the case number is low in both cases and therefore has to be regarded carefully.

Ioro et al. showed in two cadaveric studies that the descending genicular artery gives a very reliable and constant source of perfusion for the harvested graft in terms of dimensions and that it can be harvested with a skin paddle [19, 20]. Additionally, the graft can also be harvested as an osteochondral graft.

Due to the similar anatomy of the proximal scaphoid pole to the convex surface of the medial femoral trochlea, it was possible to replace the proximal pole entirely using this graft if required with a union rate of 94% [18]. Improvement of wrist pain was seen in all patients, while the wrist range of motion and the SL-angle remained similar to the preoperative state [3, 17].

In the present study, there was a slight improvement of the average SL-angle which corresponds to the study of Buerger et al. who also observed an average improvement of 6° at final follow-up [3, 17]. As Capito and Higgins pointed out, a slight oversizing of the reconstructed proximal pole (“scaphoid overstuffing”) might address the imminent risk of carpal malalignment [5]. Kalb et al. observed the spontaneous formation of a new ligamentous structure in some cases [24]. Yet, the question about the effects on intercarpal relationships and any possible advantage in stopping the progression of radiocarpal osteoarthritis remains.

Despite these reported high union rates, it remains debatable today whether a vascularized bone graft is favorable in most cases. On the one hand, an overview by Rancy et al. showed that there is no consensus about which modality is best to predict proximal pole vascularity, and that there is a lack of clear evidence that vascularized bone grafts outmatch non-vascularized bone grafts [35]. The first issue could be addressed with the above-mentioned treatment algorithm described by Schmidle et al. [42]. For the present retrospective study, there were not enough histological data on hand to evaluate a correlation with MRI data and intraoperative findings. The decision to use a vascularized MFC graft was mostly based on gadolinium-enhanced MRI scans. This approach has recently also become a body of growing debate. A missing perfusion in the MRI does not exclude bony healing [24]. Recent studies imply that proximal pole vascularity may not distinctively predict bone union, and the distal pole, as an engine for creeping substitution, may be of higher importance to predict bone healing in scaphoid non-union [34]. This study cannot provide any information on the possibility of treating histologically defined proximal pole avascular necrosis with non-vascularized bone grafts with this survey, since there was no control group.

The reported union rate of 95% was achieved with different fixation methods of the bone graft. Two non-unions occurred, one with a K-wire fixation and one with a palmar locking plate. Recent studies by Quadlbauer et al. showed a higher union rate in stabilization of scaphoid waist non-unions with two headless compression screws or a plate compared to stabilization with only one compression screw. This study cannot confirm or disprove these results, since the majority of fixations were conducted with K-wires in our population [32, 33]. The authors used the palmar locking plate for difficult bony fixations or reduction of advanced instabilities. Mehling et al. demonstrated good clinical and radiological results with palmar locking plates in difficult pathologies of the scaphoid, but also observed that most patients will require hardware removal and therefore recommended it as a rescue option [28].

Failed previous screw fixation was associated with a lower union rate (66.7%) in our population. This might originate in the combined volar and dorsal approach that was necessary in this subgroup, rather than in the previous screw fixation itself.

The MFC graft was favored by our team throughout the last years due to its good and consistent perfusion in the harvesting area, the good quality of cancellous bone and the possibility to harvest a rather big graft if necessary, as demonstrated by Iorio et al. [19]. In addition, the graft is relatively easy to harvest with some experience and convenient to shape, since it is free and not locally pedicled.

Further, as also hinted previously by Rodriguez-Vegas et al., the authors can confirm a very moderate donor site morbidity, which meets the demands of relatively young patients [37]. This is a major advantage of the MFC graft compared to the iliac crest graft which shows a significant donor site morbidity as previously stated.

The authors only conducted a microsurgical attachment of the pedicled artery and left the veins untouched. This approach differs from the method earlier described by Bürger et al. [4]. The authors did not see any problems using this modified method which reduced the operating time while achieving a high union rate. During harvesting the graft, the authors always try to achieve a minimal pedicle length of 4–5 cm to avoid tensioning of the revascularization site. By using an osteochondral graft, a good possibility of replacing the complete proximal pole exists if necessary which can lead to a union rate of 100% comparable to the results in Buerger and Higgins’ population [3, 17].

Despite the good clinical results in this study, complications occurred. However, they were rather not linked to the graft, but the surgery itself. Implant irritations healed uneventfully after removal. One delayed wound healed without surgical treatment, one venous bleeding was successfully stopped surgically and one infection showed further unproblematic bone union after implant removal, antibiotic and V.A.C therapy. The last patient suffered from severe decrease of thumb motion which had to be treated with surgical tenolysis and tendon transfer 7 months postoperatively.

There are some limitations of this study. It is a retrospective review and there is no randomization or control group. Therefore, the authors cannot conclude if a vascularized bone graft or an avascular bone graft leads to similar or better results. Also, the question whether one graft is superior to another cannot be answered. Additionally, different surgical approaches and implants were used which might distort the results. Nevertheless, the MFC graft showed good clinical results and gives the option to replace the proximal scaphoid pole preventing carpal osteoarthritis and collapse in the short term.

In conclusion, this study showed that the vascularized medial femoral bone graft leads to a good functional outcome in the treatment of scaphoid non-unions. This graft provides adequate blood supply and structural stability to the scaphoid. Additionally, a proximal pole destruction can be replaced using an osteochondral graft with promising short-term results preventing carpal osteoarthritis and collapse.
